# Deskilled and Rapid Drug-Resistant Gene Detection by Centrifugal Force-Assisted Thermal Convection PCR Device

**DOI:** 10.3390/s21041225

**Published:** 2021-02-09

**Authors:** Wilfred Villariza Espulgar, Masato Saito, Kazuya Takahashi, Sakiko Ushiro, Norihisa Yamamoto, Yukihiro Akeda, Shigeto Hamaguchi, Kazunori Tomono, Eiichi Tamiya

**Affiliations:** 1 Department of Applied Physics, Graduate School of Engineering, Osaka University, 2-1 Yamadaoka, Suita, Osaka 565-0871, Japan; wilfred@ap.eng.osaka-u.ac.jp (W.V.E.); u066418a@gmail.com (K.T.); ushiro@ap.eng.osaka-u.ac.jp (S.U.); tamiya@ap.eng.osaka-u.ac.jp (E.T.); 2 Advanced Photonics and Biosensing Open Innovation Laboratory, AIST-Osaka University, Photonics Center, Osaka University, P3 Building, 2-1 Yamadaoka, Suita, Osaka 565-0871, Japan; 3 Department of Infection Control and Prevention, Graduate School of Medicine, Osaka University, 2-2 Yamadaoka, Suita, Osaka 565-0871, Japan; norihisa65@hp-infect.med.osaka-u.ac.jp (N.Y.); akeda@biken.osaka-u.ac.jp (Y.A.); hamaguchi@hp-infect.med.osaka-u.ac.jp (S.H.); tomono@hp-infect.med.osaka-u.ac.jp (K.T.); 4 Research Institute for Microbial Diseases, Osaka University, 3-1 Yamadaoka, Suita, Osaka 565-0871, Japan

**Keywords:** drug-resistant gene, IMP-type CPE, POCT, PCR, microfluidics

## Abstract

Here we report the improved Cyclo olefin polymer (COP) microfluidic chip and polymerase chain reaction (PCR) amplification system for point-of-care testing (POCT) in rapid detection of Carbapenem-resistant Enterobacteriaceae (CRE). The PCR solution and thermal cycling is controlled by the relative gravitational acceleration (7G) only and is expected to pose minimal problem in operation by non-expert users. Detection is based on identifying the presence of carbapenemase encoding gene through the corresponding fluorescence signal after amplification. For preliminary tests, the device has been demonstrated to detect *bla*_IMP-6_ from patients stool samples. From the prepared samples, 96.4 fg/µL was detected with good certainty within 15 min (~106 thermocycles,) which is significantly faster than the conventional culture plate method. Moreover, the device is expected to detect other target genes in parallel as determination of the presence of *bla*_NDM-1_ and *bla*_OXA-23_ from control samples has also been demonstrated. With the rising threat of drug-resistant bacteria in global healthcare, this technology can greatly aid the health sector by enabling the appropriate use of antibiotics, accelerating the treatment of carriers, and suppressing the spread.

## 1. Introduction

Antibiotic-resistant bacteria cause hundreds of thousands of deaths annually in the world [1]. Especially the Carbapenem-resistant Enterobacteriaceae (CRE) with threat level raised from serious in 2013 to urgent level in 2019 as reported by the US Centers for Disease Control and Prevention (CDC) [2]. Since carbapenem serves as the “antibiotics of last resort” for the broad spectrum of bacteria, coping with the infection and administering treatment is rather difficult [3,4,5]. CRE can spread within and between health facilities or can be released in the environment as treated wastewater that can contaminate lakes and streams. In Japan, an epidemiological study by the team of Prof. Tomono at Osaka University Hospital reported that 11.8% of known CRE have been identified from facility residents at some local place [6]. In other cases, it has led to nosocomial outbreaks [7]. Culture testing, which is a typical technique for conventional drug resistance diagnosis, takes 24 h or more resulting in 48–72 h processing from patient sample to final determination [8]. Further, it can be overlooked because CRE are susceptible to imipenem as a drug generally used in culture tests [9]. Therefore, timely determination cannot be executed properly making infection control difficult.

Dissemination of antibiotic resistance in bacteria is governed by the transmission of plasmid, which is a DNA molecule physically separated from the genomic DNA. They may be present in a bacterium in varying numbers which makes identification and quantification of antibiotic resistance challenging. For the gene encoding carbapenemase (carbapenem-degrading enzyme), it may be present in the chromosome or on the plasmid. In particular, the plasmid-mediated drug resistance genes are present in large numbers such as *bla*_NDM-1_, *bla*_OXA-23_, and *bla*_IMP-6_ [10], and they can be easily and rapidly transmitted not only between homologous bacteria but also between heterologous bacteria by horizontal gene transfer [11,12]. Thus, the determination whether or not a drug-resistant gene is present regardless of bacterial species is of utmost importance. To address this concern, early detection for onsite utility through POCT (point-of-care testing) device is greatly desired. This would enable the use of appropriate antibiotics, accelerate the treatment of carriers, and suppress the spread of infection. For a POCT-oriented genetic testing device, the requirements can be patterned to devices used by the WHO (World Health Organization) at the outbreak of Ebola virus in West Africa in 2014 [13]. The operation should be less than three steps and the analysis time should be less than 30 min in addition to convenience and rapidity for field-level utility.

With the emergence of new lab-on-a-chip concepts, miniaturization of multiple processes and procedures associated with micro total analyses nucleic acids has been proven influential in developing POCT devices. Miniaturization ensures higher reaction efficiency associated with mass diffusion and heat conduction and dissipation. Moreover, the reaction can be performed with a small amount of samples and reagents for decreased operation cost. However, sufficient mixing is difficult in microfluidic channels which is crucial in nucleic acid testing. Active mixing based on actuators integrated into a microchannel is possible but there is a huge drawback since the device becomes complicated and bulky with additional components. A more desirable mixing method for biological assay application is passive mixing that utilizes the geometry of the channels (serpentine or zigzag shapes), the integrated structures (ridges, slanted well, and other three-dimensional structures), or surface properties (wettability and micropore characteristics) of the channel walls [14]. However, the extent of mixing is limited by the imposed structure configuration and cannot be turned off if needed that can pose difficulties in obtaining the optimal mixing from a given device. Another type of passive mixing, that is inherent in a rotating system, is based on Coriolis force that has been reported to enable rapid fluid mixing [15]. Uniform mixing can be easily adjusted and regulated based on the rotation speed or the radial position of the device. By integrating this driving force to multi vortex mixing in a curve channel by Dean force [16], more efficient mixing for a POCT system can be realized.

For miniaturization of PCR (polymerase chain reaction), a review paper by Lee categorizes the reported studies as chamber type or continuous flow type [17]. The latter is a more preferred choice for fully integrated microfluidic devices and is more advantageous in decreasing the reaction time since the sample flows inside a microchannel that is directly placed on multiple heaters with fixed temperatures. However, typical continuous flow type PCR has a large footprint. The walls of the long microchannel require passivation to reduce the high adsorption of molecules. One solution to this is utilizing a closed-loop channel that shortens the channel length and enables circulation of the flow. We have previously reported the use of centrifugation-controlled convection (C3) flow as an alternative method for fluid flow control in a ring-structured microchannel for PCR and ELISA [18,19]. C3 flow is based on the concept of Bénard convection but substituting the centrifugal field to the gravity field. This allows the control of thermal convection flow based on the rotation speed of the configuration when the microchannels are aligned on two heaters with a certain temperature difference. However, the previous model still requires careful measurement of minute volume to perform the PCR which can pose problems when operated by non-experts.

For POCT devices, sample delivery and reagent storage are some of the key design components. This reduces user interaction and minimizes the steps to carry out the test. Thus, the user only needs to be familiar with simple operating procedure rather than multiples ones. More importantly, with this feature, precise pipetting is not necessary which is a common source of errors from operators. Several technologies are employed to integrate this functionality in centrifugation type of systems such as passive valves based on capillary force [20] and hydrophobicity of PDMS [21] for flow process control and precise aliquoting [22]. The most practical application at present from the viewpoint of simplification and miniaturization of the PCR is considered to be the “LabDisk” [23], which performs sample extraction and preconditioning, reagent supply and routing, and amplification reaction in a chip. The necessary reagent is preloaded in the chip, and at a certain rotation speed during operation, the container is opened and the reagent filled the flow channels. However, since one thermocycle of at least 80 s is required for PCR, there is still an issue in POCT application from the viewpoint of the rapidity of the reaction.

Thus, although various microfluidic devices have been developed, a PCR device having both rapidity and easy operability to meet the above-mentioned POCT traits is left unresolved. Here, we have addressed developing a POCT gene test device based on centrifugal force-assisted thermal convection. The fluid behavior in the ring-structured microchannel was analyzed. For the preliminary test, we have evaluated the performance of the centrifugal thermal convection PCR for rapid detection in patient fecal samples having carbapenem-resistant bacteria to demonstrate its practical application.

## 2. Materials and Methods

### 2.1. Chip Fabrication

**Figure 1:**
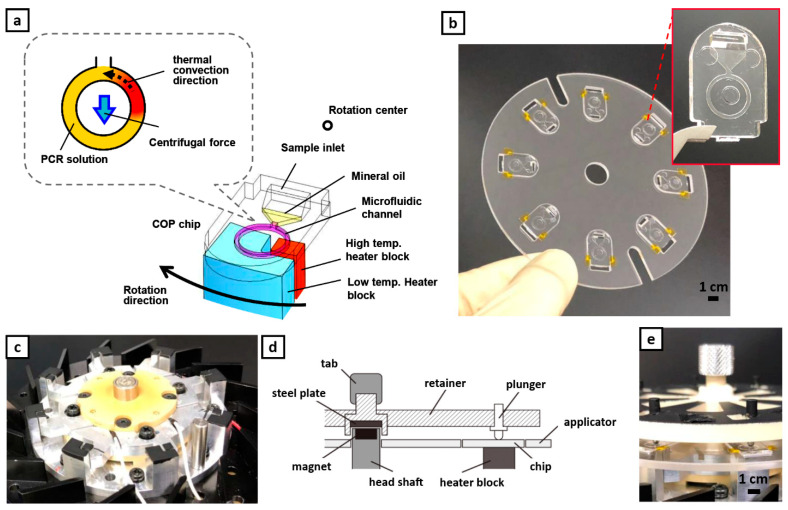
Mechanism and operation of centrifugal-assisted thermal convection polymerase chain reaction (PCR). (**a**) Schematics of alignment of the ring-structured channel to the heater block to generate the C3 flow. (**b**) Applicator holding eight plain PCR chips; all made up of Cyclo olefin polymer (COP). Inset: plain PCR chip (**c**) In-house rotating stage with integrated heaters. (**d**) Sketch illustrating how the chips are fixed to the stage. (**e**) Actual assembly of the complete set-up.

**Figure 2:**
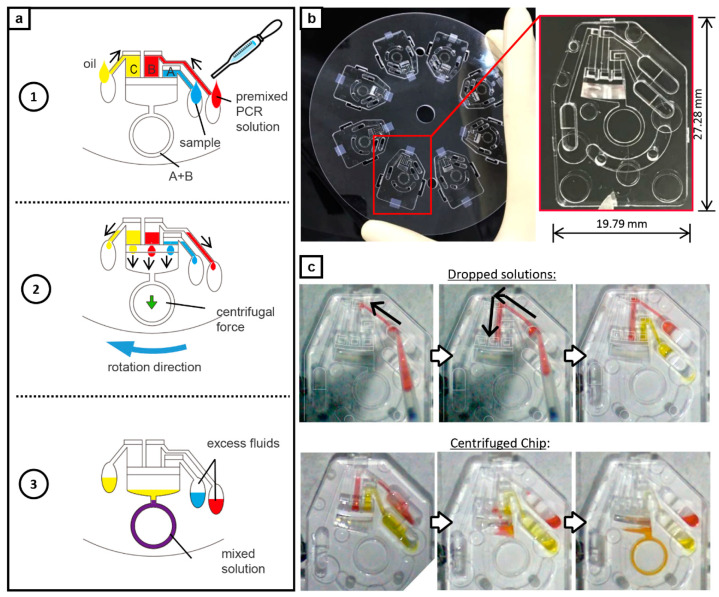
Point-of-care testing (POCT) version of the PCR chip; inclusion of capillary channels. (**a**) Schematics of fluid operation. 1: Liquids are dropped at each reservoir. Sample solution in area A, PCR solution in area B, and mineral oil in area C. 2: The solution is automatically dispensed and metered during rotation. 3: The mixture of the sample solution and the PCR solution enters the ring-structured channel for on-chip amplification while the mineral oil remains on top. (**b**) Applicator holding eight POCT PCR chips; all made up of COP. Inset shows the magnified view of the POCT PCR chip. (**c**) Fluid handling in the chip. The reagents are dropped to each respective reservoir. During centrifugation, the liquids enter the channel but only the PCR solutions enter the ring-structured channels. The oil at the top serves as cover in preventing the mixed PCR solutions from evaporating that could produce a failed amplification and an error in the readings.

The microfluidic chips are made of Cyclo olefin polymer (COP, Zeonex480, ZEON Co., Tokyo, Japan) resin which has low hygroscopicity, low self-fluorescence, high permeability, and good formability. Two types of microfluidic chips were utilized in the study; the plain chip (Figure 1) that was used for measurement calibration and optimization and the POCT chip (Figure 2) with liquid metering and delivery function that was demonstrated for practical application. In both types, the dimension of the ring-structured microchannel remains constant: 6 mm diameter, 500 µm width, and 300 µm depth, and chip thickness of 2 mm. The chips were formed by injection molding, which was outsourced from World Co. Ltd. (Takarazuka, Japan) for the plain chip and Konica Minolta, Inc. (Tokyo, Japan) for the POCT chip. To seal the molded COP chip, a COP film (188 µm in thickness, ZF14-188, ZEON Co., Tokyo, Japan) with the same outline as the chip was bonded. Primarily, the COP chip and the film were cleaned with an ultrasonic bath in IPA for 5 min and dried by blowing N_2_ gas. Contacting surfaces of the chip and the film were then treated and activated by oxygen plasma (RDC210, Yamato Scientific, Tokyo, Japan). Treatment conditions of O_2_ gas, RF time, and RF power were 100 cc, 10 s, and 75 W, respectively. Subsequently, the surfaces were adhered to one another and placed in the thermal compression apparatus (X300, SCIVAX, Kanagawa, Japan) for firm bonding. The press was operated at 133 °C with 1 MPa of pressure in −100 kPa vacuum for 30 min. After bonding, the inside walls of the microchannel were coated with 1% (w/v) Tween60 (Polyoxyethylene (20) Sorbitan Monostearate, 168-21572, Wako, Japan) for blocking and to promote a hydrophilic surface. The solution was introduced into the ring structured microchannel by centrifugation while the capillary force was utilized in the liquid delivery channel. Then, the chips were placed onto a hot plate at 100 °C to evaporate the water. This surface modification step prevents the generation of bubbles during PCR thermal cycling that can obstruct the flow.

### 2.2. Device Design and Plain Chip

Thermal cycling and fluid flow are driven in the ring-structured microchannel by spinning the device while in thermal contact with two heater blocks as shown in Figure 1a similar to the previous report [19]. From four chips, the new applicator can now hold up to eight fabricated chips (Figure 1b). Together with the microfluidic chips, the device is composed of a rotating stage, heaters, and a motor (Figure 1c). Conversely, 8 pairs of protruding heater blocks (aluminum) were integrated to the stage that conducts the heat from the ceramic heater element (MS-5, Sakaguchi E.H Voc Corp., Tokyo, Japan) to the reaction chamber upon contact. A pair of heater blocks consists of a higher temperature block (95 °C) and a lower temperature block (60 °C). The heater temperature was controlled by a digital controller (SDC 45, Azbil, Japan) while the stage rotation was controlled by a brushless motor speed control unit (NexBL US type, Oriental motor, Japan). The PCR chips were aligned to each protruding heater blocks with the aid of the applicator and were pressed down by a set of plungers as shown in Figure 1d,e. Currently, the device is capable of performing PCR simultaneously for eight samples.

### 2.3. POCT-Oriented Chip

The on-chip liquid delivery function was an added feature in developing a POCT-oriented gene testing device. The liquid delivery channel was 450 µm wide and 450 µm deep. The volumes of area A for sample solution, area B for PCR solution, and area C for mineral oil were 1.48, 2.65, and 2.65 µL, respectively. After filling the liquids in each reservoir, the chip is spun and the sample and the PCR solutions at constant volume and ratio could fill the ring-structured channel (area A + B) by centrifugation only without the need for precise pipetting. The oil remains at the top that prevents the mixed PCR solutions from evaporating that could produce a failed amplification and an error in the readings. The use of centrifugation also drives any bubbles or froth formed during amplification to buoy up which ensures no obstruction during the PCR cycle. Figure 2b shows the design and the fabricated chip. The actual fluid handling and mixing are shown in Figure 2c and Video S1.

### 2.4. Sample Preparation

#### 2.4.1. Isolates and DNA Extraction

One of the laboratory isolates in the Department of Infection Control and Prevention was used as the reference of IMP-6. The isolate (laboratory ID: 10803) was confirmed of *E. coli* with *bla*_IMP-6_ via genome sequencing. *E. coli* (BAA-2469) and *A. baumannii* (BAA-10629) were used as the other reference isolates obtained from ATCC (American Type Culture Collection). DNA was extracted with a QIAamp DNA mini kit (Qiagen, Valencia, CA, USA) according to the manufacturer’s protocol.

#### 2.4.2. Clinical Specimens

The clinical specimens used in this study were collected through surveillance in Osaka prefecture [24]. Ethical approval was obtained from the ethics committees of Osaka University, as well as collaborative institutions. A total of 178 specimens were used for this study. The specimens were cultured in M-ECC medium (CHROMagar ECC supplemented with 0.25 µg/mL meropenem and 70 µg/mL ZnSO_4_) for CRE detection.

All procedures performed to harvest the nucleic acids comply with the guidelines set by the Gene Modification Experiments Safety Committee of Osaka University.

#### 2.4.3. DNA Extraction from Clinical Fecal Samples

Clinical fecal samples were collected through surveillance in Osaka prefecture. Scraped samples were suspended into 30% glycerol solution. The sample solution was boiled for deactivation and stocked at −30 °C until examination. For on-chip PCR detection of IMP-6 gene, the fecal sample solution was prepared as 10% (*v*/*v*) in the PCR solution.

### 2.5. On-Chip PCR Assay

For the *bla*_IMP-6_ gene detection by centrifugal thermal convection PCR, DNA polymerase and optimized buffer used was GeneAceHS which was especially custom-made by Nippon Gene Co., Ltd. (Toyama, Japan). Composition of PCR solution was as follows: 1× GeneAceHS containing 5× DNA polymerase concentration compare with standard condition, 0.1% (*w*/*v*) bovine serum albumin (Sigma-Aldrich, St. Louis, MO, USA), 400 nM Fw primer: 5′-CCC ACG TAT GCA TCT GAA TTA ACA AA, 400 nM Rv primer: 5′-CCA AAC CAC TAC GTT ATC TTG AGT G, 200 nM fluorescence probe: FAM-5′-CAA GCC ACA AAT TCA TTT AGC GGA GTT AAC TAT T-TAMRA (modified version from another report [25]). Amplicon size was 159 bp. Extracted DNA sample was added as template DNA. In case of detection for clinical sample, the fecal sample solution described above was prepared as 10% (*v*/*v*) in the PCR solution. The procedures for *bla*_NDM-1_ and *bla*_OXA-23_ gene detection are detailed in the supplementary information.

A volume of 2.35 µL of prepared mixed PCR solution was injected into the sample inlet of the plain PCR chip (Figure 1a) and was transferred to the ring structured microchannel by centrifugation at 3000 rpm for 10 s. After filling the mixed PCR solution into the ring structured microchannel, 2.35 µL of mineral oil (M5904, Sigma-Aldrich, St. Louis, MO, USA) was added to the PCR chip to prevent the PCR solution from evaporating. The PCR chip was then set onto the device heater which was already set at desired temperatures and warmed up for 30 s. Then, centrifugal thermal convection PCR was performed by spinning the chips while in contact with the heater blocks. Various rotation speed (275 rpm–520 rpm/3G–11 G) and duration were tested to obtain the optimized parameters.

### 2.6. On-Chip PCR Assay with Liquid Delivery Function

To realize a POCT-oriented gene detection, the PCR chip having a liquid delivery function was used in patient fecal samples for practical application. The sample and the PCR solutions were prepared as follow; 78.7% GeneAceHS containing 5× DNA polymerase concentration compared with a standard condition, 0.15% (*w*/*v*) bovine serum albumin (Sigma-Aldrich, St. Louis, MO, USA), 630 nM Fw primer: 5′-CCC ACG TAT GCA TCT GAA TTA ACA AA, 630 nM Rv primer: 5′-CCA AAC CAC TAC GTT ATC TTG AGT G, 315 nM fluorescence probe: FAM-5′-CAA GCC ACA AAT TCA TTT AGC GGA GTT AAC TAT T-TAMRA (modified version from another report [25]). Amplicon size was 159 bp. The stocked fecal sample was diluted to be 30% with TE buffer (pH 7.0). A sufficient amount of fecal sample solution, PCR solution, and mineral oil were dropped into each inlet area. After filling the liquid delivery channel of areas A, B, and C by capillary force, the setup was rotated at 1590 rpm for 10 s to deliver the PCR solutions to the ring-structured microchannel. Temporarily, the rotation was stopped and heating was started at desired temperatures. After incubation for 30 s, centrifugal thermal convection PCR was performed for 15 min at 7G (420 rpm).

### 2.7. Measurement of Fluorescence Intensity on the PCR Chip

Fluorescence intensity on the ring structured microchannel was measured before and after amplification. The optical system, with schematics similar to a conventional confocal microscope, was constructed as described in the previous report [19]. The PCR chip was set onto a motorized stage and operated at a rate of 100 µm/s. The fluorescence signal was quantified with a data logger (TR-V550, Keyence, Osaka, Japan) through the PMT at 20 Hz sampling frequency.

For evaluating the PCR product, the presence of the target or a positive result is indicated by a measured fluorescence intensity above the threshold value of 0.088 a.u (mean fluorescence intensity of the blank) and negative otherwise after on-chip PCR process is performed.

### 2.8. Gel Shift Assay

The chip PCR product was also analyzed by the gel shift assay to verify the amplicon obtained. A volume of 1 µL of PCR solution was applied to 4% agarose gel (NuSieve 3:1, Lonza, Rockland, ME, USA) which contains EtBr for DNA staining and was run at 100 V for 15 min in TBE running buffer. A 100 bp DNA ladder (Bio-Rad, Hercules, CA, USA) was used for the DNA marker.

### 2.9. Simulation Analysis of Centrifugal Thermal Convection

Mixing behaviors which were generated by Coriolis force as secondary flow under centrifugal thermal convection in the microchannel were calculated using COMSOL Multiphysics Navier–Stokes and Heat transfer modules (COMSOL Inc, Stockholm, Sweden). The basic equations for Bénard convection were solved at steady-state and at 7G of relative gravitational acceleration. Details of the conditions were described in the previous report [19]. Additionally, time-dependent mass diffusion was calculated with and without Coriolis force. The calculation of mass diffusion was considered as the secondary fluid flow. The diffusion coefficient was set to be 1 × 10^−9^ (m^2^/s) as a typical diffusion coefficient of water. Different concentrations of the model solution (0 and 400 nM) were placed at various configurations along the circumferential direction of the microchannel. To simplify the simulation, only the ring channel was studied which is considered as an enclosed system filled with the solution.

## 3. Results and Discussion

### 3.1. Contribution of Coriolis Force to Improved Mixing Efficiency

**Figure 3:**
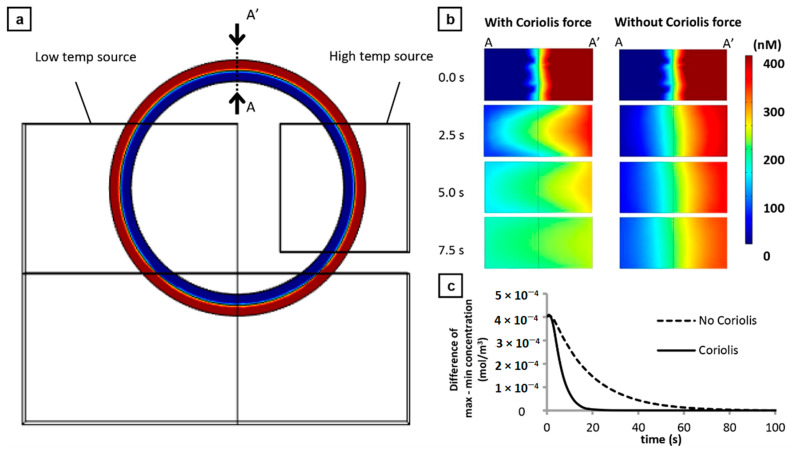
Simulation of concentration gradients in the ring-structured channel for a hypothetical case. (**a**) Model used where two solutions at different concentrations (Red—400 nM and Blue—0 were placed as concentric hollow cylinders. The concentration gradients were analyzed at region A–A’. (**b**) Time-course gradient distribution for centrifugal-assisted thermal convection (left) and free convection (right). (**c**) Graph showing the full time required for a complete mixing in the two cases studied; 4 times faster in the case with Coriolis force compared to without.

We have previously reported the generation of secondary flow in the ring-structured channel by the Coriolis force in addition to the convection flow [19]. Dean force or force due to the secondary flow in curve channels appears to have negligible effect on the mixing behavior. Additionally, the orientation of the secondary flow was dependent on the direction of the rotation (angular vector) of the holder stage; clockwise or counter-clockwise). This Coriolis-induced secondary flow is expected to enhance the passive mixing of the fluids in the microchannel which is beneficial for PCR applications. Here, a more detailed fluid behavior of the secondary flow was investigated by simulation analysis. Figure 3 illustrates a theoretical case wherein the two solutions with different concentrations (Red—400 nM and Blue—0) were placed as concentric hollow cylinders filling the ring-structured microchannel. With a high temp source value of 95 °C, low temp source value of 60 °C, 7G relative gravitational acceleration, and set at a steady-state case, the time-depended mixing and diffusion were studied at cross-section A–A’ (Figure 3a). In the presence of Coriolis force, uniform mixing was almost achieved after 7.5 s of centrifugal force-assisted thermal convection (Figure 3b). In the absence of Coriolis force or free convection and diffusion, the non-uniformity of the solution still remains after 7.5 s. Time-lapse videos (Videos S2 and S3) of the mixing behavior for both cases reveal that homogenous mixture can be achieved approximately 20 s in the presence of Coriolis force while 80 s for the free convection; four times faster (Figure 3c). Several configurations of the solution were also simulated and the same improvement in mixing was observed but the greatest value of improvement was observed in this case. More importantly, this result guarantees that the non-uniformity of the mixture will not be an issue in the operation of the device and that an efficient amplification can be achieved.

### 3.2. On-Chip PCR for bla_IMP-6_ Gene

Amplification conditions depend on the target DNA. Here, the thermal convection velocity is related to the centrifugal force and is controlled by the rotation speed. Thus, the dependency of the rotation speed to the amplification of the target gene must be determined.

**Figure 4:**
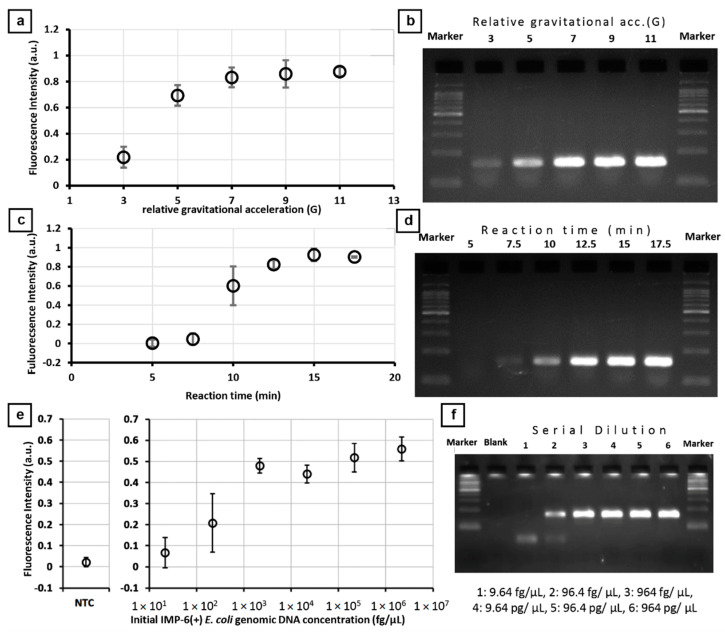
On-chip PCR to detect *bla*_IMP-6_ from the laboratory isolate 10,806 and verified with gel electrophoresis. (**a**) Fluorescence signal detected at various relative acceleration when operated at 10 min. (**b**) Corresponding gel electrophoresis analysis. (**c**) Fluorescence signal detected at various reaction times at 7G. (**d**) Corresponding gel electrophoresis analysis. (**e**) Fluorescence signals of the prepared dilution series (9.64 fg/µL to 964 pg/µL). (**f**) Corresponding gel electrophoresis analysis. All measurements were done in triplicates. Error bar represents standard deviation.

Figure 4 illustrates the optimization of operating parameters for the detection of *bla*_IMP-6_. To determine the ideal centrifugal force, the centrifugal force-assisted thermal convection PCR was performed for 10 min from 3G to 11G (*n* = 3) using 0.92 ng/µL of extracted DNA containing *bla*_IMP-6_. Figure 4a shows that the increase in the measured fluorescence intensity signal increases from 3G and starts to plateau at 7G. To verify the amplification, the products were subjected to gel electrophoresis and the specific band at 159 bp was confirmed (Figure 4b). The band density increased from 3G to 7G and then remains about the same in 9G and 11G. The increase of amplification from 3G to 7G is related to the number of thermocycles within 10 min of operation. The number of PCR reaction cycles at 5G is approximately 59 cycles (10.2 s/cycle) in 10 min which is usually already saturated in bulk PCR. This apparent decrease in reaction efficiency is associated with the adsorption of reagents to the walls of the channel and the parabolic flow velocity distribution of the main flow. For 9G and 11G, the approximate numbers of PCR reaction cycles in 10 min are 83 cycles (7.2 s/cycle) and 105 cycles (5.7 s/cycle), respectively. Aside from reaching the saturation point, a low value of fluorescence signal associated with the amplification is related to a short reaction time that could have reduced the completion of denaturing, annealing, and extension stages of the PCR. From this result, 7G is the relative gravitational acceleration set for IMP-6 where one cycle runs for 8.5 s.

The optimum reaction time was determined by varying the gene amplification duration from 5 to 17.5 min (*n* = 3) at 7G. Figure 4c reveals that the fluorescence signal starts to be noticeable at 7.5 min and significantly increases at 10 min then starts to plateau at 12.5 min. The same trend was observed to the individual products subjected to gel electrophoresis where the specific band at 159 bp was confirmed (Figure 4d). The band intensity reaches saturation at 12.5 min. The corresponding number of cycles for 5, 7.5, 10, 12.5, 15, and 17.5 min are 35.3, 52.9, 70.6, 88.2, 105.9, and 123.5 cycles, respectively. The 52.9 cycles in 7.5 min are usually saturated in bulk PCR which could have stemmed from the reduced reaction efficiency; similar to the findings in the previously optimized parameter. From this result, the reaction time used for succeeding experiments is 15 min to guarantee to obtain the maximum fluorescence possible.

A dilution series of *E. coli* purified DNA containing *bla*_IMP-6_ was prepared from 9.64 fg/µL to 964 pg/µL and on-chip PCR was performed for each concentration (*n* = 3). Figure 4e reveals that 96.4 fg/µL is the lowest concentration prepared that has a fluorescence intensity signal value greater than the negative control +3σ or the expected limit of detection based on blank determination. The same trend of an increased signal can be observed in the electrophoresis analysis results where the specific band at 159 bp was confirmed (Figure 4f). Saturated band amplifications were observed at concentrations higher than 96.4 fg/µL. Conversely, the large error bar near the detection limit is an expected consequence of the matrix effect when preparing low concentration samples manually which affects the sensitivity of the device. More importantly, no unspecific PCR products were observed from the gel shift assays which shows that the specificity is not compromised in this platform. Still, quantitative measurement is not yet possible for the system but should be competitive enough to determine the presence or absence of antibiotic-resistant genes for POCT.

Similar experiments were conducted for isolates with *bla*_NDM-1_ and *bla*_OXA-23_. The lowest concentration detected from prepared samples were 1070 fg/µL for *bla*_NDM-1_ and 293.6 fg/µL for *bla*_OXA-23_ when the system was operated at 7G for 15 min (Figure S1).

### 3.3. Agreement of On-Chip PCR Test Results with Conventional Culture Test Results

To evaluate the capability for the rapid detection of drug resistance genes of the centrifugal force-assisted convection PCR, stool samples containing *bla*_IMP-6_ were studied. A total of 178 stool samples were tested using the device and the results were compared to that of the culture tests (24 h incubation) performed at the Infection Control Department of the School of Medicine at Osaka University.

**Figure 5:**
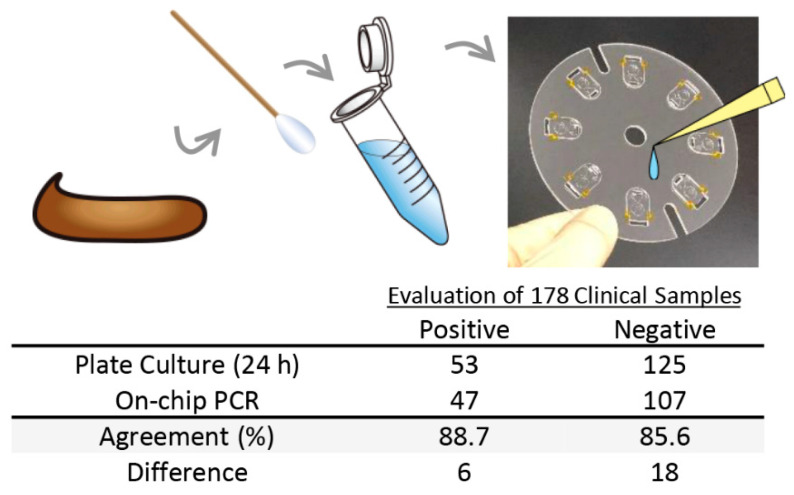
Comparison of plate culture test and on-chip PCR test for detecting Carbapenem-resistant Enterobacteriaceae (CRE) from 178 stool samples.

Figure 5 summarizes the comparison of the results. Out of the 53 positive samples, 47 samples were detected by the on-chip PCR device; 88.7% agreement. One possible reason for getting a false negative response is that the template DNA introduced in the chip was too low. Hoping to increase the sensitivity, the experiment was performed at higher stool sample concentration; from 10% to 32%. However, the result showed a negative response. Moreover, a problem arises wherein the previously determined positive sample by the on-chip PCR has been determined negative. The reaction must have been inhibited by the increase in the amount of feces and glycerol in the PCR solution. Thus, the detection sensitivity could not be improved by increasing the fecal concentration.

Aside from the presence of inhibitors and the matrix effect in getting a false negative result, another reason could be related to the non-enzymatic mechanism for carbapenem resistance of the pathogen. Even without the gene to encode the enzyme that hydrolyzes the drug, bacteria can be resistant by preventing the drug from reaching and inactivating the penicillin-binding proteins (PBPs), which are responsible for maintaining the bacterial cell wall that is essential for growth and reproduction [10]. This can be done by an active transport system to expel the drug to the periplasmic space after their entrance [26,27,28]. In the case of *E. coli*, more than 30 considered drug transporter genes exist which can be the focus in the future study [29] to expand the system’s utility. The pathogen may also have a mutation and had lost certain outer membrane porins where drugs enter resulting in diminished permeability and decreased uptake [30,31]. These non-enzymatic mechanisms cannot be detected currently by this system and will require a separate test to work in parallel for more accurate detection of the presence of CRE.

It is indeed ideal to minimize the false negatives, especially in medical institutions’ operation. To reduce the false negatives by this method, an extraction and preconcentration step of drug resistance genes in the stool sample should be considered in the future development of the system. However, it should be pointed out that despite the lack of a special sample preparation method, more than 85% of the positive samples from culture tests could be detected by the system demonstrating its high sensitivity and reliability for rapid in situ diagnosis.

Out of 125 negative samples, 107 samples were accurately detected by the on-chip PCR device; 85.6% agreement. The 18 samples not detected by the device was further tested with gel electrophoresis. The specific band was confirmed and 17 samples resulted to be positive. These 17 false negative results from the culture tests probably occurred because the bacteria are already eliminated during the sample preparation and processing. Furthermore, a false positive result from the on-chip PCR is considered better than a false negative reading from the viewpoint of preventing the spread of infection. Currently, the fluorescence measurement of the on-chip PCR device is endpoint detection. False positives can be avoided by having a real-time measurement of the fluorescence signal during amplification which can be another direction in developing the system.

### 3.4. Demonstration of POCT Application

The POCT version of the fluidic chip (Figure 2c, Video S1) enables automatic metering and delivery of the sample solution, PCR solution, and mineral oil into the ring-structured channel by simply spinning the applicator without the need for careful measurement of minute volumes. Figure S2 shows that the excess fluids are prevented from entering the reaction channel by simply placing absorbent cotton at the inlet reservoir.

**Figure 6:**
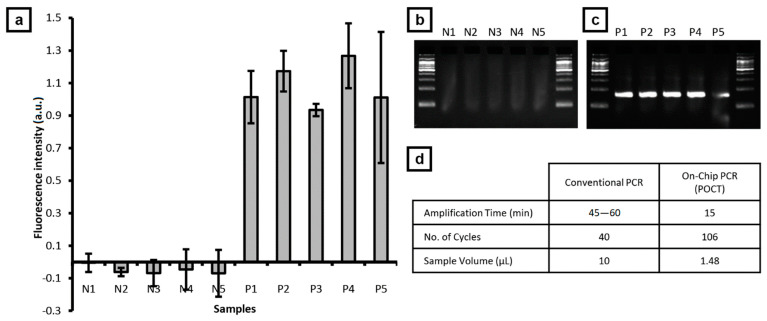
On-chip PCR of stool samples using POCT chip. (**a**) Fluorescence signal detected from test samples that are validated with gel electrophoresis for (**b**) five negative and (**c**) five positive samples containing *bla*_IMP-6_. (**d**) Comparison of POCT on-chip PCR with conventional PCR.

Five stool samples, that were prepared in triplicates, were randomly selected from each of *bla*_IMP-6_ positive and negative samples identified by both centrifugal thermal convection PCR and culture test. Figure 6 shows the summary of the test results of the on-chip PCR and validated by gel electrophoresis based on the specific band at 159 bp. No specific amplification product was observed in the negative sample (Figure 6b) whereas a specific amplification product was observed in the positive sample (Figure 6c). This shows that the detection performance is preserved in the POCT version at faster amplification time and reduced sample volume requirement (Figure 6d) and is expected to be useful in rapid detection for onsite utility.

## 4. Conclusions

Rapid detection of the presence of an antibiotic-resistant gene was successfully demonstrated using centrifugal force-assisted convection PCR; estimated less than 20 min of operation from sample preparation to readout for *bla*_IMP-6_. The performance of the device for the practical application was made evident by detecting the presence of *bla*_IMP-6_ from stool samples which are the most difficult and time-consuming clinical samples to process. Multi-drug resistant gene detection can be performed with the device if warranted as detections of *bla*_NDM-1_ and *bla*_OXA-23_ were already performed. With the integration of the automatic metering and delivery function to the PCR chip, the POCT utility of the system has been achieved. This enables ease in operation while still capable of rapid detection of the carbapenem-resistance gene at high sensitivity. By this system, testings can be significantly advanced and quickened, which will be beneficial for therapy and for monitoring the spread of the resistant organism or resistant genes throughout the medical institutions and the community. This system can provide a significant technical advancement for a faster turnaround of patient sampling to final determination leading to improved health care. In addition, the system is versatile to detect other nucleic acids by simply changing the primers for the identification of other infectious diseases (i.e., nCoV, tuberculosis, malaria, dengue, etc.) that will be valuable in low-resource settings.

## Data Availability

Data available on request due to privacy restrictions.

## Supplementary Materials

The following are available online at https://www.mdpi.com/1424-8220/21/4/1225/s1, Figure S1: On-chip PCR to detect *bla*_NDM-1_ genes from *E. coli* (BAA-2469) and *bla*_OXA-23_ genes from *A. baumannii* (BAA-10629), Figure S2: Inclusion of absorbent cotton to prevent excess fluid from entering the PCR channel, Video S1: Fluid handling and mixing, Video S2: Mixing behavior in the presence of Coriolis force (spinning), Video S3: Mixing behavior in free convection.
