# Supplementary material for: Deskilled and Rapid Drug-Resistant Gene Detection by Centrifugal Force-Assisted Thermal Convection PCR Device
Source: Sensors (Basel). 2021 Feb 9;21(4):1225. doi: 10.3390/s21041225 (PMC7916093; doi:10.3390/s21041225)
Supplement: Supplementary file 1 [file sensors-21-01225-s001.zip › sensors-1072992 - final supplementary.pdf]

Supplementary Materials

Deskilled and Rapid Drug-Resistant Gene Detection by Centrifugal Force-Assisted Thermal Convection PCR Device

Wilfred Villariza Espulgar [1], Masato Saito [1,2,*], Kazuya Takahashi [1], Sakiko Ushiro [1], Norihisa Yamamoto [3,4], Yukihiro Akeda [3,4], Shigeto Hamaguchi [3,4], Kazunori Tomono [3] and Eiichi Tamiya [1,2]

1 Department of Applied Physics, Graduate School of Engineering, Osaka University, 2-1 Yamadaoka, Suita, Osaka 565-0871, Japan; wilfred@ap.eng.osaka-u.ac.jp (W.V.E.); u066418a@gmail.com (K.T.); ushiro@ap.eng.osaka-u.ac.jp (S.U.); tamiya@ap.eng.osaka-u.ac.jp (E.T.)

2 Advanced Photonics and Biosensing Open Innovation Laboratory, AIST-Osaka University, Photonics Center, Osaka University, P3 Building, 2-1 Yamadaoka, Suita, Osaka 565-0871, Japan

3 Department of Infection Control and Prevention, Graduate School of Medicine, Osaka University, 2-2 Yamadaoka, Suita, Osaka 565-0871, Japan; norihisa65@hp-infect.med.osaka-u.ac.jp (N.Y.); akeda@biken.osaka-u.ac.jp (Y.A.); hamaguchi@hp-infect.med.osaka-u.ac.jp (S.H.); tomono@hp-infect.med.osaka-u.ac.jp (K.T.)

4 Research Institute for Microbial Diseases, Osaka University, 3-1 Yamadaoka, Suita, Osaka 565-0871, Japan

* Correspondence: saitomasato@ap.eng.osaka-u.ac.jp

1. For the *bla*$_{NDM-1}$ Gene Detection

Composition of PCR solution was as follow; 1× Ampdirect Plus (241-08800-98, Shimadzu, Kyoto, Japan), 0.25 U/μL SpeedSTAR HS DNA Polymerase (RR070B, Takara Bio, Shiga, Japan), 0.1% (w/v) bovine serum albumin (Sigma-Aldrich, St. Louis, MO, USA), 0.01% (v/v) Polyvinylpyrrolidone (PVP, Sigma-Aldrich, St. Louis, MO, USA), 800 nM Fw primer: 5′-CGC AAC ACA GCC TGA CTT T, 800 nM Rv primer: 5′-TCG ATC CCA ACG GTG ATA TT, 200 nM fluorescence probe: FAM-5′-CAA CTT TGG CCC GCT CAA GGT ATT T-BHQ1. Amplicon size was 127 bp. Total DNA was extracted from *bla*$_{NDM-1}$ positive *E. coli* (ATCC: BAA-2469), and was used for template DNA.

2. For the *bla*$_{OXA-23}$ Gene Detection

Composition of PCR solution was as follow; 1× Ampdirect Plus, 0.25 U/μL SpeedSTAR HS DNA Polymerase, 0.1% (w/v) bovine serum albumin, 400 nM Fw primer: 5′-GAC ACT AGG AGA AGC CAT GAA G, 400 nM Rv primer: 5′-CAG CAT TAC CGA AAC CAA TAC G, 200 nM fluorescence probe: FAM-5′-CCA GTC TAT CAG GAA CTT GCG CGA-BHQ1. Amplicon size was 116 bp. Total DNA was extracted from *bla*$_{OXA-23}$ positive *A. baumannii* (BAA-10629) and was used for template DNA.

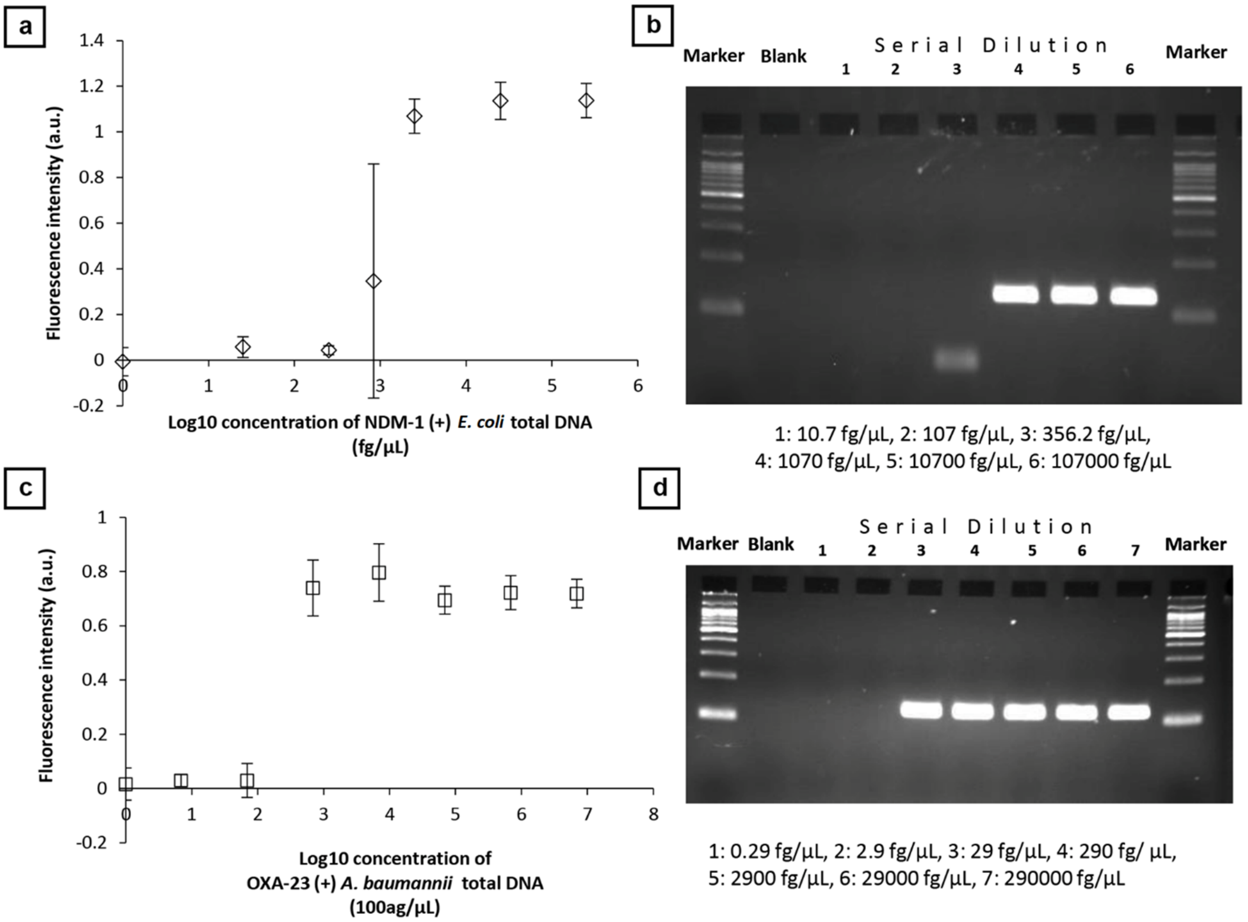

Figure S1. On-chip PCR to detect (**a**) *bla*$_{NDM-1}$ genes from *E. coli* (BAA-2469) and (**c**) *bla*$_{OXA-23}$ genes from *A. baumannii* (BAA-10629) purified DNA and verified with gel electrophoresis (**b** and **d**). Error bar represents standard deviation ($n = 3$).

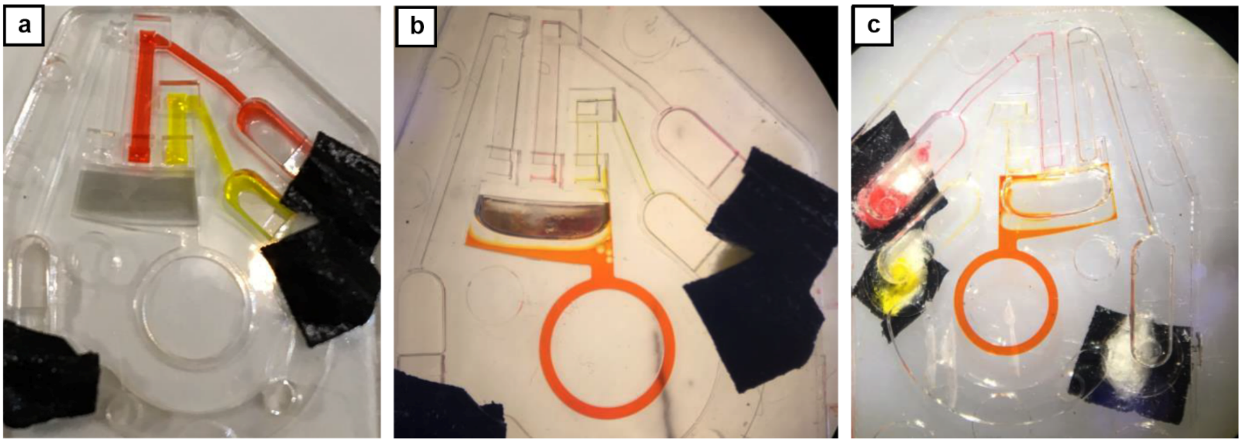

Figure S2. Inclusion of absorbent cotton to prevent excess fluid from entering the PCR channel. (**a**) Dropping the solutions to respective chambers. (**b**) Image of the chip after centrifugation. (**c**) Backside view showing the cotton containing the excess liquids.
